# Characterization of death receptor 3‐dependent aortic changes during inflammatory arthritis

**DOI:** 10.1002/prp2.240

**Published:** 2016-06-10

**Authors:** Jessica O. Williams, Eddie C. Y. Wang, Derek Lang, Anwen S. Williams

**Affiliations:** ^1^ Division of Infection and ImmunityCardiff University School of MedicineCardiffUnited Kingdom; ^2^ Division of Medical EducationCardiff University School of MedicineCardiffUnited Kingdom

**Keywords:** Death receptor 3, inflammatory arthritis, inflammatory ingress, vascular constriction

## Abstract

Murine collagen‐induced arthritis (mCIA) is characterized by decreased vascular constriction responses and increased MMP‐9. Here, we describe additional histological alterations within the aorta and surrounding perivascular adipose tissue (PVAT), study the role of PVAT in constriction response, and investigate the potential involvement of death receptor 3 (DR3). mCIA was induced in wild‐type (WT) and DR3^−/−^ mice with nonimmunized, age‐matched controls. Vascular function was determined in isolated aortic rings ±PVAT, using isometric tension myography, in response to cumulative serotonin concentrations. Cellular expression of F4/80 (macrophages), Ly6G (neutrophils), DR3, and MMP‐9 was determined using immunohistochemistry. In WTs, arthritis‐induced vascular dysfunction was associated with increased F4/80+ macrophages and increased DR3 expression in the aorta and PVAT. MMP‐9 was also up‐regulated in PVAT, but did not correlate with alterations of PVAT intact constriction. DR3^−/−^ mice inherently showed increased leukocyte numbers and MMP‐9 expression in the PVAT, but retained the same nonarthritic constriction response as DR3WT mice ±PVAT. Arthritic DR3^−/−^ mice had a worsened constriction response than DR3WT and showed an influx of neutrophils to the aorta and PVAT. Macrophage numbers were also up‐regulated in DR3^−/−^ PVAT. Despite this influx, PVAT intact DR3^−/−^ constriction responses were restored to the same level as DR3WT. Impaired vascular constriction in inflammatory arthritis occurs independently of total MMP‐9 levels, but correlates with macrophage and neutrophil ingress. Ablating DR3 worsens the associated vasculature dysfunction, however, DR3^−/−^ PVAT is able to protect the aorta against aberrant vasoconstriction caused in this model.

**Abbreviations**
CV: cardiovascular
CVD: cardiovascular disease
DR3: death receptor 3
DR3^−/−^: death receptor 3 knockout
DR3WT: death receptor 3 wild type
fT_reg_: fat resident regulatory T cell
MMP‐9: matrix metalloproteinase 9
mAB: monoclonal antibody
mCIA: murine collagen‐induced arthritis
NO: nitric oxide
PVAT: perivascular adipose tissue
RA: rheumatoid arthritis
T_reg_: regulatory T cells
SPM: specialized pro‐resolving mediators
WT: wild type

## Introduction

Rheumatoid arthritis (RA) is a risk factor for cardiovascular (CV) disease (CVD). Indeed, RA patients are twice as likely to suffer a CV death than age‐matched healthy individuals (Solomon et al. 2006); (Roman and Salmon 2007). Coronary artery disease is one of the most common causes of death in RA patients and its risk is evident even before a clinical RA diagnosis (Maradit‐Kremers et al. 2005). The cause of increased risk of CVD for RA patients is not well defined. However, an association between RA, high lipid profiles, and underlying levels of systemic inflammation have recently been identified (Myasoedova et al. 2010).

A role for innate immune cells, such as macrophages and neutrophils, in the pathogenesis and progression of RA is long standing and well defined (Koch et al. 1994); (Kinne et al. 2000). Whether the presence of these cells impacts on the vasculature contributing to associated CVD remains in question, even though the presence of these cells in the aortic vessel wall and surrounding perivascular adipose tissue (PVAT) has been postulated since 1986 (Jonasson et al. 1986). It is known that the number of macrophages is able to increase in PVAT and is dependent on factors such as age, nutritional status, and local environment (Skilton et al. 2009). Thus, we describe the impact of systemic inflammation on these cellular populations in both the vasculature and the surrounding PVAT.

To mechanistically investigate these tissues in human disease is difficult, if not, impossible. Thus, this “proof‐of‐concept” study uses an appropriate surrogate animal model. With regard to looking at early systemic inflammatory changes in relation to RA, murine collagen‐induced arthritis (mCIA) is a well described and characterized model (Hegen et al. 2008). We have previously used the mCIA model to characterize vascular responses in early RA, describing decreased constriction responses associated with increased aortic matrix metalloproteinase 9 (MMP‐9) (Reynolds et al. 2012). MMP‐9 is a key mediator involved in degradation of the extracellular matrix during the remodeling process that occurs “normally” in the vasculature. However, it is often inappropriate remodeling, driven by increase in proteins such as MMP‐9 and decrease in their inhibitors that underpins CV pathology (Janssens and Lijnen 2005). Given the reported role of immune cells in the production of MMP‐9 in a dysfunctional vasculature (Rizas and Ippagunta 2009); (Zhang et al. 2011) and the potential function of such cells in RA, further characterization of the vascular phenotype in mCIA would provide insight into the association between RA and CVD.

An important signaling molecule, death receptor 3 (DR3), has been linked to the differentiation of macrophages to the foam cells that populate atherosclerotic plaque (McLaren et al. 2010), neutrophil accumulation in arthritic joints, and increased MMP‐9 production in inflammatory arthritis (Wang et al. 2014). DR3 is the closest relative to TNFR1, the receptor for TNF*α*. It can signal both cell death via its intracellular death domain and cell survival via activation of transcription factor NF‐*κβ* (Kitson et al. 1996); (Marsters et al. 1996). DR3 has one known TNFSF ligand – TNF like protein 1 A (TL1A) (Migone et al. 2002), a close relative of TNF*α* (Jin et al. 2007), a master regulator of inflammation that is up‐regulated in RA (Feldmann and Maini 2003). Interestingly, in the antigen‐induced model of arthritis, DR3 knockout (DR3^−/−^) mice exhibit a decrease in the articular population of neutrophils, compared to wild type (WT) (Wang et al. 2014). Whether DR3 modulates or contributes to the vascular dysfunction in RA is an obvious area for further study.

The aim of this study was to explore the causes of decreased vascular constriction in mCIA. The presence of inflammatory cells in both the aortic vessel wall and PVAT of arthritic and normal tissues were determined. Focus was directed to macrophages, neutrophils, and total MMP‐9 production. The impact of ablation of DR3 expression on these measurements was also investigated. For the first time, we establish a relationship between experimental arthritis onset and the nature and extent of immune cell ingress into the aortic vessel wall and PVAT, and describe protection from vascular dysfunction in the absence of DR3.

## Materials and Methods

### Animals

Male mice (8 weeks old) were used for all experiments. WT DBA/1 mice were acquired from Harlan, UK. DBA/1 DR3 knockout (DR3^−/−^) and appropriately age‐matched DBA/1 DR3 WT mice (DR3WT) were sourced from an in‐house breeding colony, generated by DR3^het^ × DR3^het^ crossing. The DBA DR3^−/−^ colony was produced through backcrossing C57Bl/6^het^ mice with DBA/1 WT mice for seven generations. All animal care and experimental procedures complied with the United Kingdom Animals (Scientific Procedures) Act 1986 and were under the authority of Home Office Project Licence (30/2928).

### Induction of mCIA

mCIA was induced as previously described (Nowell et al. 2009; Reynolds et al. 2012). In brief, mice were immunized on two occasions, 21 days apart, with identical 100 *μ*L intradermal injections of an emulsion containing 1 mg/mL type II chicken sternal collagen (Sigma, Dorset, UK) and 2.5 mg/mL Freund's complete adjuvant. Temgesic (0.4 mg/mL) was administered ad libitum via the drinking water on day 20 before arthritis onset and was continued until the end of each experiment.

### Assessment of arthritis

Joint swelling was assessed (under isoflurane) daily following immunization on day 21 as previously described (Reynolds et al. 2012). Hind paw swelling was recorded for both paws using an analog micrometer. Each paw was also scored 0–5 (Table 1) dependent on arthritis onset and a combined paw score was used to determine total severity; mild arthritis (1–5), moderate arthritis (6–9), and severe arthritis (10–14).

**Table 1:** Paw scoring system

Paw score	Pathological features
0	Normal
1	Mild/moderate erythema and swelling (single toes effected with no other swelling)
2	Severe swelling encompassing whole paw
3	Severe swelling encompassing the whole paw, and toes beginning to become effected
4	Whole paw/ankle/toes swollen
5	Deformed paw/ankylosis

### Collection of experimental samples

At experimental end point, defined as a combined paw score of up to 5 (mild arthritis) or at day 29 for DR3WT versus DR3^−/−^ experiments, mice were killed by inhalation of CO_2_. Subsequently, the aorta was exposed and vented in the abdominal region. The left ventricle was then perfused with 500 *μ*L of physiological Krebs solution (mmol/L: NaCl 10.92, KCl 2.68, KH_2_PO_4_ 1.78, MgSO_4_.7H_2_O 2.49, NaHCO_3_ 25.10, glucose 10.99, CaCl_2_.2H_2_0 1.98). The thoracic aorta was dissected from the animal with PVAT intact and immediately placed in iced Krebs solution for myography. Alternatively, tissues isolated for use in histology and QPCR were fixed/stored in 70% (w/v) ethanol (minimum of 1 week at 4°C) or “RNA later” (overnight at 4°C followed by long term storage at −20°C), respectively. Not all tissues from all animals were used for all experiments.

### Determination of vascular constriction

Myography was used to investigate vascular constriction to serotonin (5‐HT) in isolated sections of thoracic aorta in the absence (−) or presence (+) of PVAT. Such experiments have been previously described (Cai et al. 2005; Reynolds et al. 2012). Aortic rings (2 mm thick) from nonarthritic control and mild arthritic mice were bathed in physiological Krebs solution at 37°C and gassed with 95% O_2_/5% CO_2_. Each ring was set to a baseline tension of 5 mN. Following equilibration, rings were exposed to a “wake up response” of 60 mmol/L potassium Krebs (mmol/L: NaCl 39.36, KCl 59.83, KH_2_PO_4_ 1.18, MgSO_4_.7H_2_O 2.49, NaHCO_3_ 25.10, glucose 10.99, CaCl_2_.2H_2_0 1.98). Once constriction had reached a plateau, tissues were washed and allowed to return to baseline tension. Cumulative concentration responses to 5‐HT (1 nmol/L–10 mmol/L) were then carried out. Tissue contractile response was measured using MyoDak software and analyzed using MyoData and was calculated as developed tension in mN.

### Histological evaluation of the aortic vessel and PVAT

Following fixing, samples were processed in the Shandon tissue processor before paraffin wax blocks were formed. Sections, 8 *μ*m in thickness, were cut using a microtome and placed onto Superfrost+ slides and kept at 70°C overnight. Each section was rehydrated, stained using hematoxylin (1 min) and eosin (30 sec), before being dehydrated and mounted using DPX. Images at ×10 magnification were acquired using a Leica microscope and camera and were analyzed using Image J and Carestream software.

### Protein identification by immunohistochemistry

Immunohistochemistry was used to visualize protein markers (F4/80 (macrophages), Ly6G (neutrophils), DR3, and total MMP‐9) within the aortic vessel wall and surrounding PVAT. A standard protocol was used for each, with specific antibodies for individual targets. In brief, sections were rehydrated and blocked for endogenous peroxidase, avidin, and biotin activity. Rat anti‐mouse F4/80 (Abcam, Cambridge, UK) and Ly6G (BD Pharmigen, Oxford, UK) was used at 2 and 1 *μ*g/mL, respectively with a secondary rabbit anti‐rat IgG antibody (Vector Labs, Peterborough, UK). For both DR3 and MMP‐9 expression, specific biotynylated goat anti‐mouse antibodies (R&D Systems, Abingdon, UK) were used at 2 and 15 *μ*g/mL, respectively. Appropriate isotypes were used for each antibody. Positive protein identification was determined using a streptavidin–HRP conjugate and DAB chromogen in all cases. Sections were then counterstained with hematoxylin before being dehydrated and mounted with DPX. High‐power images at ×40 magnification were taken with a Leica microscope and camera and analyzed using Photoshop.

All immunohistochemical analysis was performed blind. Total cells were counted using a computer program called Living Image, in which, each cell was detected by “edge” (the computer identifies changes in color to find the edges of each cell nuclei). The computer automatically detects each cell nuclei in a given area. Percentage of positive staining refers to the staining intensity overall, within the given region.

### Statistical analysis

Data are expressed as mean ± SEM, *n* ≥ 4 for all experiments. Not all animal samples were used for each experiment. Constriction response data are expressed as developed tension in mN and was fitted to a sigmoidal curve using GraphPad Prism. Rmax and EC50 values were calculated for each response curve to determine the concentration that produced maximal and half maximal constriction. All data were compared using Student's *t*‐tests and a significant difference was considered as *P* < 0.05.

## Results

### The presence of PVAT impacts both normal and arthritic vascular constriction responses

**Figure 1:**
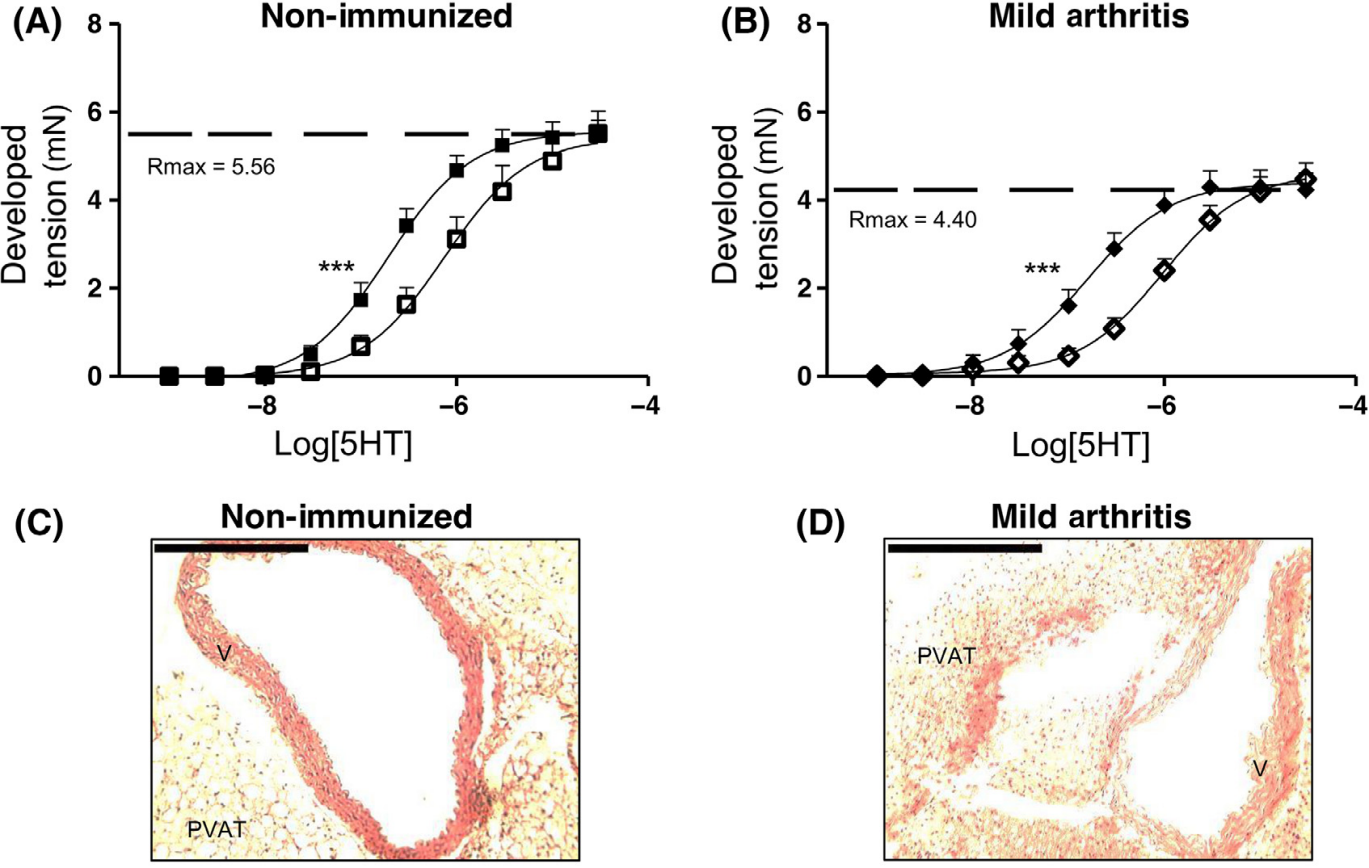
The impact of wild‐type arthritis on constriction response ± perivascular adipose tissue (PVAT). Constriction responses to 5HT were determined using myography in aortic rings of non‐immunized control aorta compared with arthritic aorta; +PVAT (Arthritic *N* = 13, control *N* = 8) and –PVAT (Arthritic *N* = 14, control *N* = 8). (A) and (B) show comparison of EC_50_ within 5HT response curves; non‐immunized +PVAT versus –PVAT and mild arthritic aorta +PVAT versus −PVAT respectively. Representative images show hematoxylin and eosin staining of aortic vessel wall and PVAT of non‐immunized controls (C) and arthritic aorta (D). Both maximal constriction and EC_50_ were compared using an unpaired *t*‐test. ***P < 0.001. SEM bars are shown in one direction. Images are taken at ×20 magnification. Scale bars represent 0.25 *μ*m. V represents aortic vessel wall and PVAT represents surrounding perivascular fat. (A, B) Key; Non‐Immunized, +PVAT (□), –PVAT (■). Mild Arthritis +PVAT (◊), −PVAT (♦).

As previously described by Reynolds et al. (2012), we showed significantly (*P* < 0.001) depressed maximal constriction responses of arthritic aortic tissue ‐PVAT following exposure to 5‐HT (4.40 ± 0.05 mN, *n* = 14), in comparison to nonimmunized control tissue (5.56 ± 0.06 mN, *n* = 8). No differences in EC_50_ values were observed (Fig. 1).

For the first time, the impact of the presence of PVAT on the arthritic vascular constriction response was also investigated. In both arthritic (*n* = 13) and nonimmunized control (*n* = 8) aorta, PVAT caused significant (*P* < 0.001) dextral shifts in the constriction responses to 5‐HT, altering EC_50_ (−5.98 ± 0.02 mN vs. −6.13 ± 0.04 mN) (Fig. 1). Maximal constriction was not impacted by PVAT and remained significantly (*P* < 0.05) decreased in arthritic tissue compared to nonimmunized controls (4.96 ± 0.06 mN vs. 5.43 ± 0.10 mN).

Decreased constriction response following mCIA onset was further evaluated to determine inflammatory context of the aortic vessel wall and PVAT. Hematoxylin and eosin staining highlighted the different structure of the vessel wall and PVAT following arthritis onset (Fig. 1). Despite the total cell number within the PVAT remaining unchanged, the composition of the PVAT between nonimmunized and arthritic animals appears different. Nonimmunized PVAT would seem to consist of white adipocytes, with a single cell nucleus and a large adipocyte vesicle. However, within arthritic PVAT, cells look densely packed and have smaller fat‐filled vesicles, more indicative of a brown fat phenotype.

### Inflammatory cell infiltration increases in both the aortic vessel wall and PVAT in inflammatory arthritis

**Figure 2:**
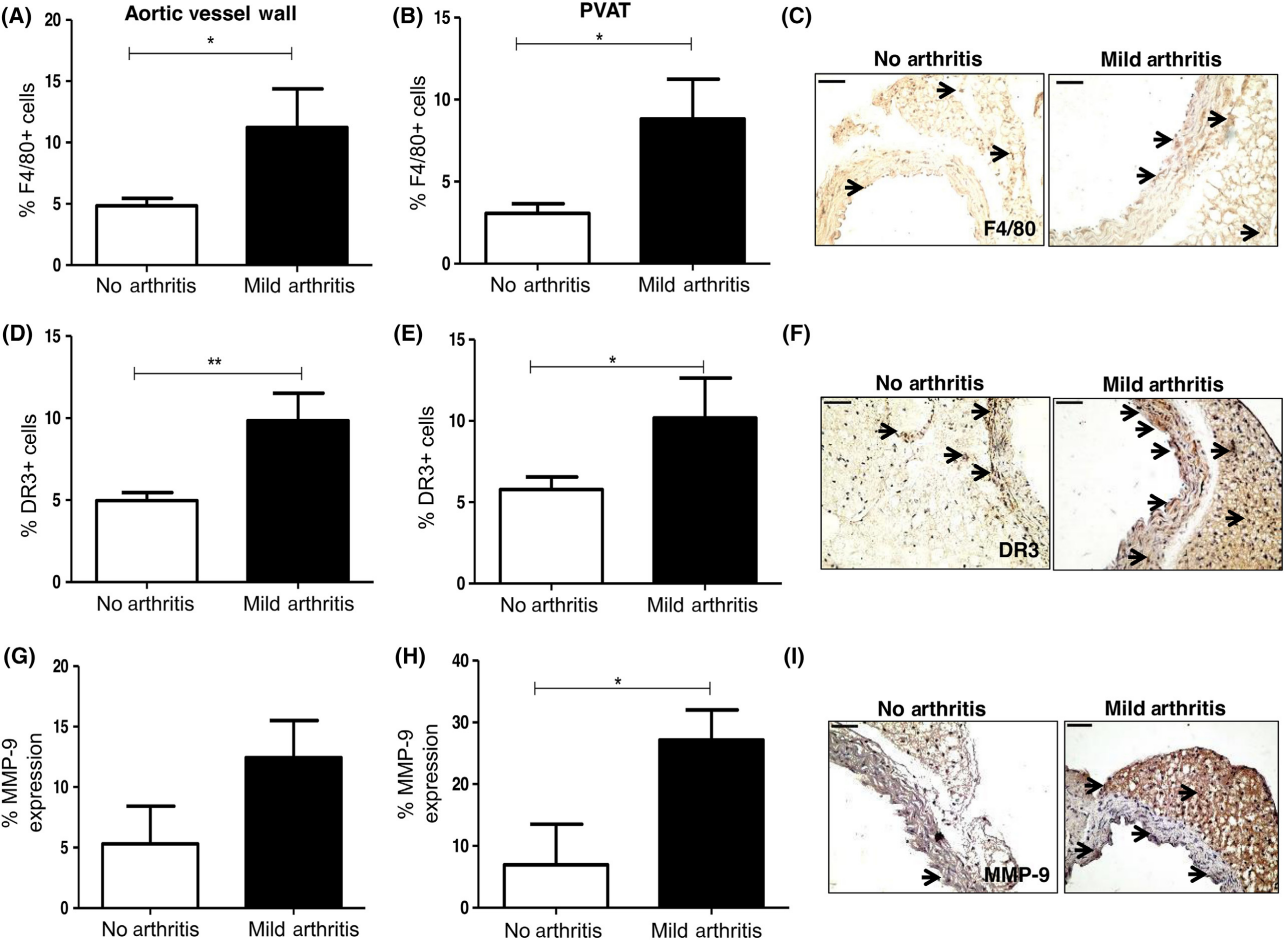
Inflammation‐associated changes in the aortic vessel wall and surrounding perivascular adipose tissue (PVAT). Following the onset of murine collagen‐induced arthritis macrophage ingress (A) and DR3 expression (D) are significantly increased in the aortic vessel wall, compared to non‐arthritic controls, however, MMP‐9 expression remains unchanged (G). In the PVAT, macrophage (B) and DR3 expression (E) along with MMP‐9 expression (H) was increased in arthritic animals in comparison to non‐arthritic controls. Representative images show examples of F4/80 (G), DR3 (H), and MMP‐9 (I) staining in nonarthritic and arthritic aorta and PVAT. Images are taken at ×40 magnification and scale bars represent 0.2 *μ*m. Unpaired *t*‐tests were used for all shown comparisons. *P < 0.05, **P < 0.01. Error bars represent SEM. N number was at least eight in arthritic group compared with a minimum of four controls.

Characteristic signs of inflammation were observed in the aortic vessel wall following induction of mCIA (Fig. 2). In mild mCIA, the number of macrophages, as estimated by F4/80 signal, was significantly (*P* < 0.05) increased when compared to nonimmunized controls (11.2 ± 3.2%, *n* = 15 vs. 4.8 ± 0.6%, *n* = 8; Fig. 2A). For the first time, we show that this macrophage ingress was associated with an increased DR3 signal, in the absence of atheroma, during inflammatory arthritis (9.8 ± 1.7%, *n* = 8 vs. 5.0 ± 0.5%, *n* = 13) (*P* < 0.01, Fig. 2). However, neither MMP‐9 (12.5 ± 3.1%, *n* = 12 vs. 5.3 ± 3.1%, *n* = 4), total leukocyte number (10424 ± 1328 cells/mm^2^, *n* = 8 vs. 11438 ± 608 cells/mm^2^, *n* = 21), nor neutrophil numbers as estimated by Ly6G signal (0.6 ± 0.2%, *n* = 10 vs. 1.9 ± 0.9%, *n* = 4) were significantly increased following arthritis onset (data not shown).

PVAT was analyzed separate from the aortic vessel wall to investigate whether unique differences could be associated with the dextral shift in vascular constriction response. A similar inflammatory profile was observed in the surrounding PVAT as to that seen in the aortic vessel wall. F4/80+ macrophage staining was significantly (*P* < 0.05) increased in mild disease when compared to nonimmunized controls (8.8 ± 2.4%, *n* = 15 vs. 3.1 ± 0.6%, *n* = 8) and this was again mirrored by significantly (*P* < 0.05) increased DR3 expression in mild arthritis (10.2 ± 2.5%, *n* = 14 vs. 5.8 ± 0.8%, *n* = 8). Unlike the aorta, a significant (*P* < 0.05) increase in total MMP‐9 was observed in the arthritic PVAT (27.2 ± 4.9%, *n* = 11 vs. 7.0 ± 6.6%, *n* = 4) (Fig. 2F). Total leukocyte numbers (6856 ± 743 cells/mm^2^, *n* = 8 vs. 5821 ± 453 cells/mm^2^, *n* = 21) and neutrophils (%Ly6G+: 1.8 ± 1.1%, *n* = 9 vs. 1.6 ± 0.9%, *n* = 4) remained unchanged when comparing mild arthritic PVAT with nonimmunized PVAT (data not shown).

### DR3 expression influences cellular infiltrate in PVAT, but not vascular constriction, in the absence of arthritis

**Figure 3:**
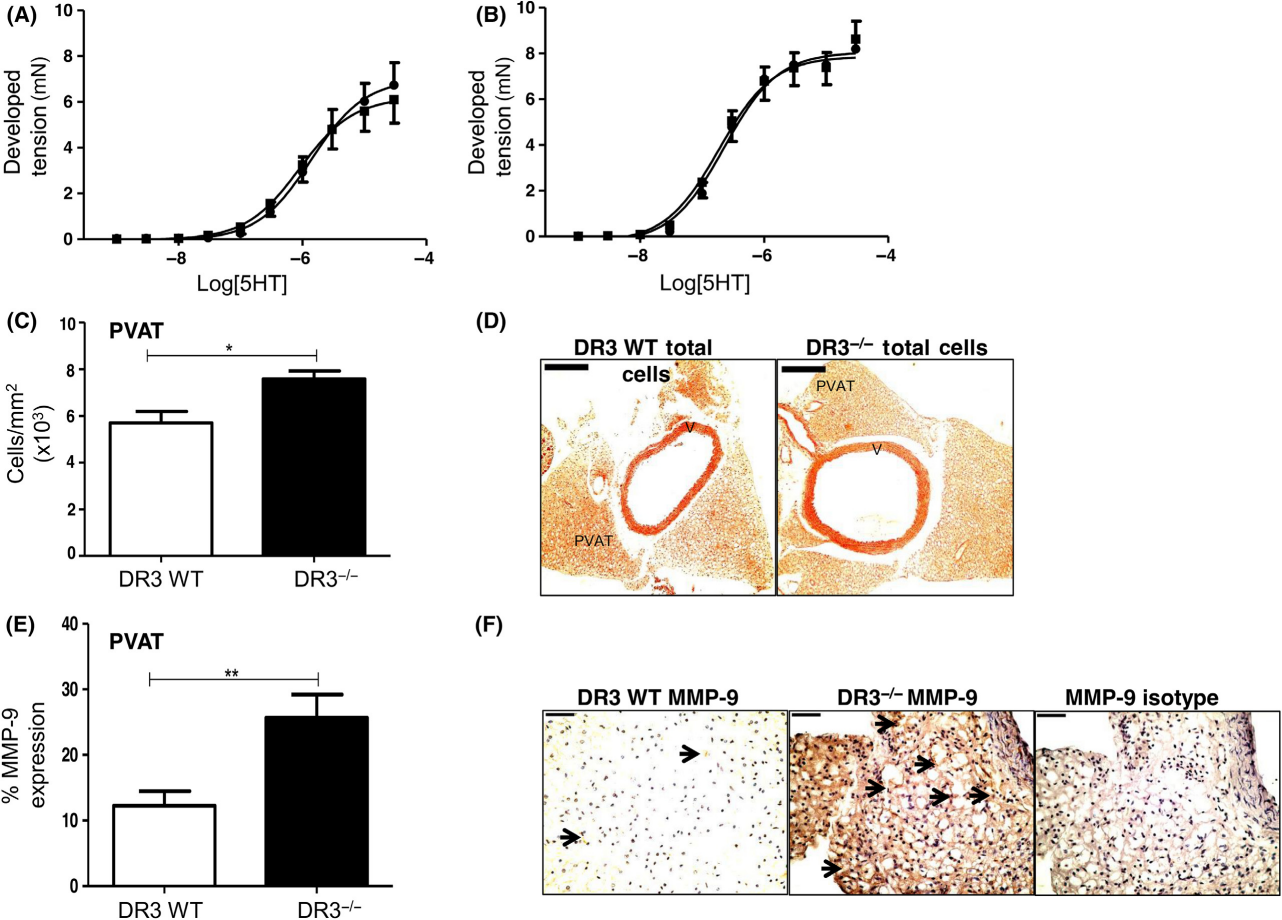
The impact of DR3 and vascular constriction and inflammation in the aorta and perivascular adipose tissue (PVAT). Ablation of DR3 has no inherent impact on vascular constriction +PVAT (A) or –PVAT (B). The ablation of DR3 increases total leukocyte number in the PVAT (C), as seen by representative hematoxylin and eosin staining between DR3 wild type (WT) and DR3^−/−^ (D). Total MMP‐9 production (E) is also increased in the PVAT of DR3^−/−^. Representative images are provided of MMP‐9 immunohistochemistry in DR3 WT and DR3^−/−^, with suitable isotype control (F). Unpaired *t*‐tests were used for all shown comparisons. *P < 0.05, **P < 0.01. SEM bars are shown in one direction. Images are taken at ×20 (Total Cells) and ×40 (MMP‐9) magnification. Scale bars represent 0.2 *μ*m. N numbers were at least 9 for DR3^−/−^ and was compared to a minimum of 10 DR3 WT. (A, B) Key; DR3WT (■), DR3^−/−^ (•).

The association between altered constriction responses and an increase in DR3 expression in both the aorta and surrounding PVAT (Fig. 2) suggested that the DR3/TL1A pathway could be involved in regulating vascular function. This was tested by exploring DR3^−/−^ tissue using the same readouts. The ablation of DR3 had no impact on the vascular constriction response seen to 5‐HT. Rmax was similar in DR3^−/−^ and DR3WT mice +PVAT (2.21 ± 0.79 mN, *n* = 10 vs. 2.22 ± 0.86 mN, *n* = 10) and –PVAT (3.82 ± 1.13 mN, *n* = 10 vs. 3.71 ± 1.14 mN, *n* = 10) (Fig. 3). A dextral shift in the constriction response curve to 5‐HT was again observed in the presence of PVAT. This data indicate that DR3 expression does not intrinsically alter the responses of the aortic vasculature.

These results were mirrored by minimal impact on cellular infiltrate within the aortic vessel wall (Fig. 3). DR3^−/−^ mice showed similar numbers of F4/80 +  macrophages (3.6 ± 0.8%, *n* = 9 vs. 5.2 ± 0.8%, *n* = 11), MMP‐9 expression (13.6 ± 1.7%, *n* = 16 vs. 11.3 ± 2.8%, *n* = 14), and total leukocytes (10968 ± 307 cells/mm^2^, *n* = 19 vs. 12142 ± 606 cells/mm^2^, *n* = 17) in comparison to DR3WT mice.

More interestingly, the PVAT of the healthy DR3^−/−^ mice had significantly (*P* < 0.05) higher total leukocyte counts (7578 ± 346 cells/mm^2^, *n* = 19) compared to DR3WT controls (5695 ± 489 cells/mm^2^, *n* = 17). Total MMP‐9 production was also significantly (*P* < 0.01) increased in the PVAT of DR3^−/−^ (25.7 ± 3.5%, *n* = 19), when compared to DR3WT, mice (12.3 ± 2.2%, *n* = 17) (Fig. 3). Macrophage numbers (%F4/80+: 1.9 ± 0.5%, *n* = 9 vs. 2.8 ± 0.6%, *n* = 11) remained unchanged, when DR3^−/−^ mice were compared with DR3WT. This suggests that DR3, in the absence of inflammation, intrinsically regulate cellular infiltration specifically into the PVAT, although this does not influence the vascular constriction responses.

### In the absence of DR3, inflammation alters the cellular infiltrate into aorta but not PVAT

**Figure 4:**
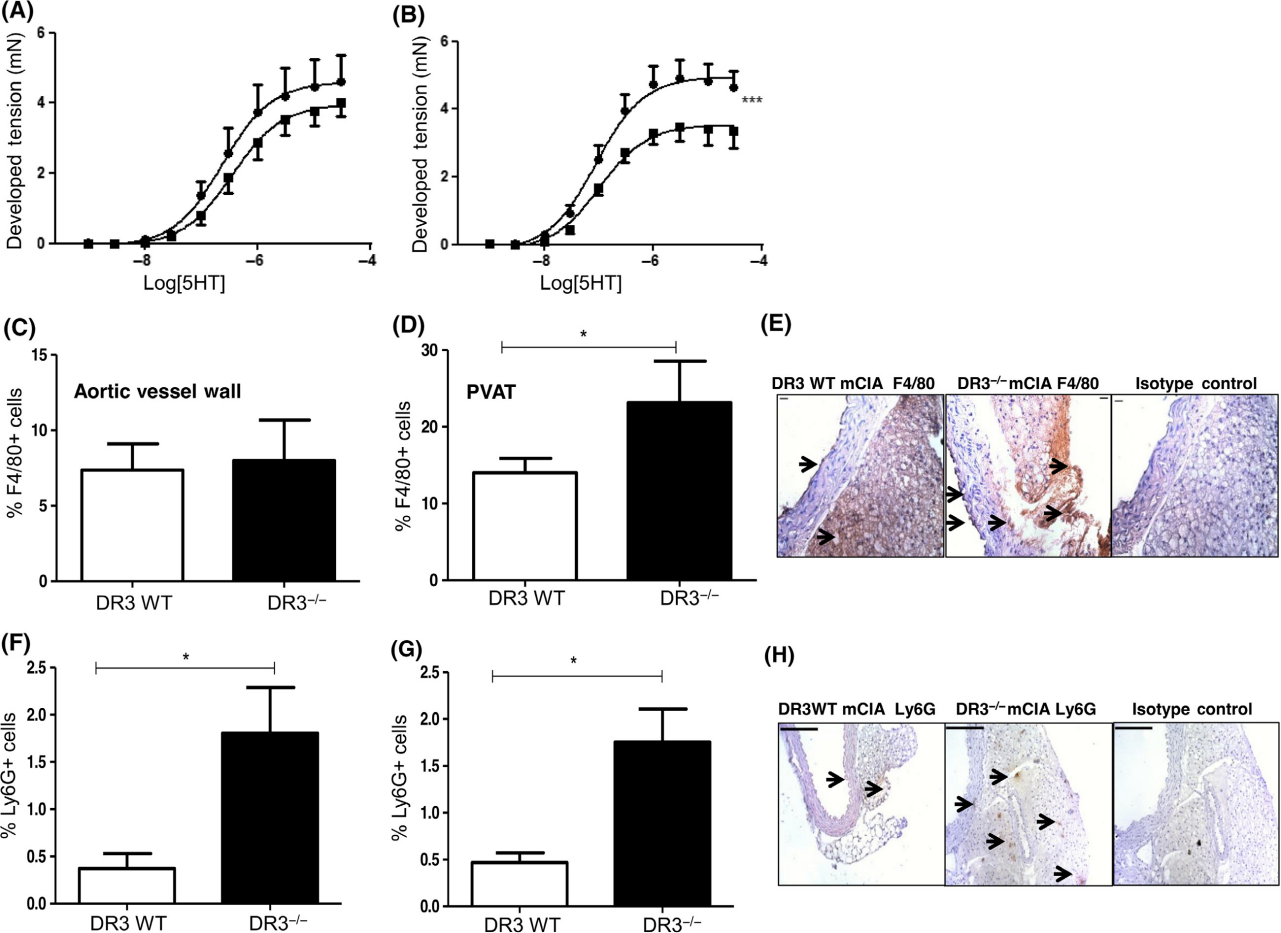
The role of DR3 in murine collagen‐induced arthritis‐associated vascular changes. Vascular constriction responses to 5HT were compared for DR3 wild type (WT) (•) versus DR3^−/−^ (▪), + perivascular adipose tissue (PVAT) (A) and –PVAT (B). Macrophage number remained consistent between DR3 WT and DR3^−/−^ in the aortic vessel wall (C), however, were increased in the PVAT of DR3^−/−^ (D). Representative images (E) are shown of positive macrophage staining in the aortic vessel wall and PVAT, with appropriate isotype control. The number of neutrophils was increased in both the aortic vessel wall (F) and the PVAT (G) of DR3^−/−^ in comparison to DR3 WT. Representative images (H) show positive neutrophil staining in aortic vessel wall and PVAT, with appropriate isotype control. Unpaired *t*‐tests were used for all shown comparisons. *P < 0.05, ***P < 0.001. Error bars represent SEM and are shown unidirectional. Images are taken at ×40 magnification (Macrophages) and ×20 (Neutrophils). Scale bars represent 0.2 and 0.25 *μ*m, respectively. A minimum of six arthritic DR3^−/−^ was compared with at least five arthritic DR3 WT.

Experiments were then performed on DR3^−/−^ mice in which CIA had been induced. Following arthritis onset, DR3^−/−^ LY6G+ neutrophil signal in the aorta was significantly (*P* < 0.05) increased in comparison with arthritic DR3WT mice (1.8 ± 0.5%, *n* = 4 vs. 0.4 ± 0.2, *n* = 6) (Fig. 4). However, F4/80+ macrophage numbers (7.4 ± 1.7%, *n* = 8 vs. 8.0 ± 2.7%, *n* = 6), MMP‐9 production (14.4 ± 2.8%, *n* = 8 vs. 11.7 ± 3.2%, *n* = 5), and total leukocyte number (2088 ± 478 cells/mm^2^, *n* = 8 vs. 2042 ± 348 cells/mm^2^, *n* = 5) remained unchanged.

Characteristic signs of inflammation were seen in the PVAT with a significant (*P* < 0.05) increase in neutrophil number observed in arthritic DR3^−/−^ compared with DR3WT mice (1.8 ± 0.4%, *n* = 6 vs. 0.5 ± 0.1%, *n* = 5) (Fig. 4). Macrophage number was also significantly (*P* < 0.01) increased in arthritic DR3^−/−^ (%F4/80+: 27.2 ± 4.4%, *n* = 5) when compared to arthritic DR3WT mice (14.0 ± 1.9%, *n* = 8) (Fig. 4). Total leukocyte numbers (1425 ± 245 cells/mm^2^, *n* = 8 vs. 1726 ± 348 cells/mm^2^, *n* = 5) and MMP‐9 (34.4 ± 2.8%, *n* = 7 vs. 18.2 ± 6.5%, *n* = 5) production remained unchanged.

### During inflammatory arthritis, DR3^−/−^ tissue exhibit impaired vascular responses only in the absence of PVAT

Following arthritis onset and exposure to 5‐HT, Rmax values for DR3^−/−^ arthritic aortic tissue −PVAT (3.53 ± 0.16 mN, *n* = 10) were significantly depressed (*P* < 0.001) in comparison to arthritic DR3WT tissues (4.94 ± 0.21 mN, *n* = 10). No differences in EC_50_ values were observed (Fig. 4). Intriguingly, the presence of PVAT restored the DR3^−/−^ maximal constriction to the same level as DR3WT (4.59 ± 0.34 mN, *n* = 10 vs. 3.96 ± 0.20 mN, *n* = 10).

## Discussion

The increased risks to health associated with systemic inflammatory diseases such as RA and CVD are well established, however, the underlying mechanisms linking these disorders to clinical events is poorly understood. Here, we show that systemic inflammation leads to an impaired vascular constriction response that is impacted by the presence of PVAT. DR3 is involved in the vascular constriction response and PVAT modulates this response. Finally, all of these processes are associated with alterations in the cellular infiltrate into both the aorta and PVAT.

Vascular contractile dysfunction in mCIA in the absence of PVAT has been described previously (Reynolds et al. 2012), but this study demonstrates for the first time that PVAT caused a general dextral shift in response to 5‐HT in both control and mCIA settings, with maximal responses being similar to those without PVAT (Fig. 1). While the implication is that PVAT does not alter its function in a mCIA setting, this builds on an increasing body of evidence that PVAT is not just an inert fat store providing physical protection to the vasculature, but that it plays a major role in the modulation of vascular constriction responses (Löhn et al. 2002); (Verlohren et al. 2004). PVAT produces factors that modulate both vasoconstriction and relaxation (Gao et al. 2006), behaving differently according to its immediate environment and responding to the onset of local inflammation and hypoxia (Greenstein et al. 2009).

We further investigated the impact of DR3 expression in this system because signaling through this TNFRSF member has been shown to modulate 5‐HT expression in the brain (Twohig et al. 2010), as well as being essential for the progression of inflammatory arthritis in the periphery. In mCIA, animals treated with antagonistic anti‐TL1A mAb (Bull et al. 2008) and those on a TL1A^−/−^ background show ameliorated disease (Wang et al. 2013), while the addition of TL1A worsens clinical and histopathological scores (Zhang et al. 2009). This is also true in Antigen Induced Arthritis (AIA), where DR3^−/−^ mice are being protected from bone erosions (Bull et al. 2008) and cartilage destruction (Wang et al. 2014). Addition of TL1A also exacerbates AIA in a dose‐ and DR3‐dependent manner (Bull et al. 2008). This essential requirement in murine arthritis models is supported by data from human disease where increased TL1A concentrations are found in the serum and synovial fluid of RA patients, and are linked with autoantibody production (Zhang et al. 2009).

Our data show that the role of DR3 in vascular constriction seems more ambiguous. In the absence of inflammation, DR3^−/−^ constriction responses were comparable with DR3WT with or without PVAT (Fig. 3), suggesting no role in constriction in a healthy setting. In contrast, although DR3^−/−^ mice were protected from arthritic pathology, their constriction response without PVAT was far worse than their DR3WT counterparts, indicating that DR3 was generally protective in the vasculature during systemic inflammation. Even more intriguingly, the addition of DR3^−/−^ PVAT returned DR3^−/−^ constriction to DR3WT levels (Fig. 4), demonstrating that DR3^−/−^ PVAT has a vasoconstricting effect. This is similar to WT PVAT function following the establishment of obesity where production of oxidative and inflammatory factors increase contractility (Agabiti‐Rosei et al. 2014), but opposite to WT PVAT's vasorelaxant role in an inflammatory setting.

It is clear from this data that the constitution of the tissues surrounding the vasculature (PVAT and aortic wall) will likely determine its functional responsiveness. As a potential effector molecule capable of degrading stromal tissue and impacting on contractility through its gelatinase and collagenase activity, we first chose to study MMP‐9. Primarily, MMP‐9 was chosen because of previous reports that described; firstly, increased levels associated with vascular dysfunction in mCIA (Reynolds et al. 2012), secondly, the capacity of DR3 signaling to trigger MMP‐9 release from macrophages in vitro (Kang et al. 2005); (Collins et al. 2015), and finally, the reduced levels of MMP‐9 observed in the absence of DR3 in AIA (Wang et al. 2014). MMP‐9 levels were increased in PVAT, but not the aortic wall, of WT mice undergoing mCIA (Fig. 2). Surprisingly, MMP‐9 was also up‐regulated in the PVAT of healthy DR3^−/−^ mice. Defining the underlying reasons behind this will require further study, as many cell types can produce MMP‐9; ranging from macrophages, neutrophils, and fibroblasts (Wang et al. 2014) to adipocytes (Bouloumié et al. 2001), mast cells (Kanbe et al. 1999), and mesenchymal stem cells. Many of these cells, such as, macrophages and neutrophils can also express DR3. There was, however, no correlation between levels of MMP‐9, either in the PVAT or aortic wall, and alterations in the constriction response irrespective of the presence or absence of DR3, suggesting that MMP‐9 has no dominant role in regulating vascular dysfunction.

Perhaps most significantly with regard to function, inflammation and/or DR3 expression altered the cellular composition of either the aortic vessel wall or PVAT. In the absence of inflammation, while leukocyte numbers in the aortic wall remained unchanged, DR3^−/−^ PVAT showed increased total leukocyte counts, suggesting that DR3 intrinsically controls the cellular content within the PVAT. A more detailed analysis will be required to determine: firstly, which cell populations were increased (they cannot be macrophages as the F4/80 signal remained unchanged) and secondly, which DR3‐dependent mechanism(s) drive this homeostatic function. As its name suggests, DR3 can induce apoptosis in vitro (Kitson et al. 1996); (Marsters et al. 1996), and in vivo (Wang et al. 2001); (Al‐Lamki et al. 2008) but the majority of its physiological functions are associated with proliferation, differentiation, and effector cytokine release (Bamias et al. 2003); (Fang et al. 2008); (Meylan et al. 2008); (Pappu et al. 2008); (Takedatsu et al. 2008); (Wang 2012); (Bull et al. 2008) including the production of chemokines (Kang et al. 2005); (Wang et al. 2014) that may attract or release different subsets of cells. All of these processes can alter the cellular composition of a tissue. Irrespective, this is the first time a homeostatic role for DR3 on cell content of a nonimmune tissue has been described.

Systemic inflammation itself, regardless of DR3 expression, was associated with alterations in tissue composition. Thus, while the number of total cells within both the aortic vessel wall and PVAT were unchanged, increased F4/80+ expression suggests rising macrophage numbers, but a concomitant decrease in as yet unidentified cell subsets within the tissue. Ablation of DR3 in an inflammatory setting caused a further significant increase in neutrophil numbers within the aortic cell wall. Whether these changes are enough in themselves to drive the observed vascular dysfunctions is a question for debate. However, it has already been established that macrophages have multiple functions in the vasculature, whether they are resident macrophages in the aorta (Gerrity 1981) carrying out “normal” functions such as tissue remodeling (Fantin et al. 2010), or infiltrating inflammatory macrophages that produce a complex mixture of chemokines and adhesion molecules that induce inflammation (Wellen and Hotamisligil 2003); (Permana et al. 2006). Although more work would be required to determine the specific macrophage phenotypes within the PVAT, and how changes to these phenotypes would impact the vascular constriction response, we can speculate on the type of macrophages that may be present. As we see a protective role of PVAT in DR3^−/−^, where numbers of macrophages are increased, we would expect to see an increase in M2 – anti‐inflammatory macrophages (Murray and Wynn 2011). While this does not necessarily mean there will not be any M1 – pro‐inflammatory macrophages present, it is likely that the balance has switched toward being M2 dominant. The relationship between M1 and M2 macrophages in PVAT has recently been evaluated. Ruan et al. (2015) showed how a decrease in M1, coupled with an increase in M2 ameliorates vascular injury. Moreover, it is also possible that increased production of IL‐10 by M2 macrophages underlies the protective effect of the PVAT (Weisser et al. 2013). IL‐10 is a potent anti‐inflammatory protein, and while knocking out the IL‐10 gene has no impact on vascular constriction in healthy mice, following an inflammatory stimuli ablation of IL‐10 exacerbates vascular contractile dysfunction (Gunnett et al. 1999). Similarly, experimental data from human studies has shown that neutrophils can produce an endothelium‐derived relaxing factor‐like product, causing vasorelaxation (Mehta et al. 1991) and both cell types can be induced to produce nitric oxide.

One other subset of immune cells is worthy of mention in the context of DR3, their presence in adipose tissue, involvement in controlling inflammation, and therefore the potential to influence vascular responses. Fat‐resident regulatory T cell (fT_reg_) modulate the inflammatory state of adipose tissue and are reduced in murine models of obesity (Feuerer 2009) where a clear decrease in vascular function is evident (Stapleton et al. 2008). Interestingly, DR3 is constitutively expressed on T_reg_ (Twohig et al. 2012), and signaling through TL1A promotes their proliferation (Meylan et al. 2011); (Shih et al. 2011); (Taraban et al. 2011). Indeed, T_reg_ expansion driven by hexameric TL1A can protect against allergic lung inflammation and has been trailed in models of transplant tolerance (Khan et al. 2013). It is tempting to speculate that DR3^−/−^ PVAT would have impaired fT_reg_ numbers or function, which could impact on the ability of this tissue to regulate vascular constriction in an inflammatory setting.

Despite there being no currently published data suggesting a link between DR3 expression and the production of specialized pro‐resolving mediators (SPM), we postulate the potential for the two systems to be linked. Focusing specifically on a subset of SPM known as lipoxins, that play a critical role in resolving local tissue inflammation and maintaining homeostasis, it is possible that they promote macrophage‐driven uptake of neutrophils found in inflammatory sites (Godson et al. 2000). We postulate that this signal may be lost in our DR3^−/−^ colony since in both the aortic vessel wall and PVAT we see increased neutrophil numbers in comparison to DR3 WT. Further studies are required to determine the role of this potential link.

In summary, the data presented here suggest that aortic vascular dysfunction is driven by changes in the inflammatory status of the vasculature and PVAT. Alterations in numbers of macrophages and neutrophils in both regions are associated with the observed contractile changes. The ablation of DR3 is protective against arthritis, but does not diminish the associated systemic effects seen in the vasculature. The importance of the PVAT is highlighted as despite inflammatory changes, this tissue is protective of normal vascular function. However, considerably more work is required to understand the underlying mechanisms controlling these processes.

## Author Contributions

J. O. W., D. L., and A. S. W. participated in research design; J. O. W. and A. S. W. conducted experiments; E. C. Y. W. contributed new reagents or analytical tools; J. O. W. performed data analysis; J. O. W., D. L., A. S. W., and E. C. Y. W. wrote or contributed to the writing of the manuscript.

## Disclosures

None declared.
